# MmpL3 as a Target for the Treatment of Drug-Resistant Nontuberculous Mycobacterial Infections

**DOI:** 10.3389/fmicb.2018.01547

**Published:** 2018-07-10

**Authors:** Wei Li, Amira Yazidi, Amitkumar N. Pandya, Pooja Hegde, Weiwei Tong, Vinicius Calado Nogueira de Moura, E. Jeffrey North, Jurgen Sygusch, Mary Jackson

**Affiliations:** ^1^Mycobacteria Research Laboratories, Department of Microbiology, Immunology and Pathology, Colorado State University, Fort Collins, CO, United States; ^2^Biochimie et Médecine Moléculaire, Université de Montréal, Montréal, QC, Canada; ^3^Groupe d'Étude des Protéines Membranaires, Université de Montréal, Montréal, QC, Canada; ^4^Department of Pharmacy Sciences, School of Pharmacy and Health Professions, Creighton University, Omaha, NE, United States

**Keywords:** *Mycobacterium abscessus*, nontuberculous mycobacteria, MmpL3, mycolic acids, drug development

## Abstract

Nontuberculous mycobacterial (NTM) pulmonary infections are emerging as a global health problem and pose a threat to susceptible individuals with structural or functional lung conditions such as cystic fibrosis, chronic obstructive pulmonary disease and bronchiectasis. *Mycobacterium avium* complex (MAC) and *Mycobacterium abscessus* complex (MABSC) species account for 70–95% of the pulmonary NTM infections worldwide. Treatment options for these pathogens are limited, involve lengthy multidrug regimens of 12–18 months with parenteral and oral drugs, and their outcome is often suboptimal. Development of new drugs and improved regimens to treat NTM infections are thus greatly needed. In the last 2 years, the screening of compound libraries against *M. abscessus* in culture has led to the discovery of a number of different chemotypes that target MmpL3, an essential inner membrane transporter involved in the export of the building blocks of the outer membrane of all mycobacteria known as the mycolic acids. This perspective reflects on the therapeutic potential of MmpL3 in *Mycobacterium tuberculosis* and NTM and the possible reasons underlying the outstanding promiscuity of this target. It further analyzes the physiological and structural factors that may account for the apparent looser structure-activity relationship of some of these compound series against *M. tuberculosis* compared to NTM.

## Introduction

The prevalence of pulmonary nontuberculous mycobacterial (NTM) infections caused by *Mycobacterium avium* complex (MAC) and *Mycobacterium abscessus* complex (MABSC) species is increasing worldwide and poses a particular threat to susceptible individuals with structural or functional lung conditions such as cystic fibrosis (CF), chronic obstructive pulmonary disease, and bronchiectasis (Park and Olivier, 2015; Parkins and Floto, 2015; Bryant et al., 2016; Floto et al., 2016; Martiniano et al., 2016). Treatment options for NTM pulmonary infections involve lengthy (12–18 months) combination regimens with antibiotics that lack bactericidal activity and are associated with significant toxicity. For pulmonary MAC, the recommended treatment includes a macrolide, rifamycin, and ethambutol to which intravenous amikacin may be added. Treatment of pulmonary MABSC typically consists of an oral macrolide in conjunction with intravenous or inhaled amikacin, and one or more of the following drugs: intravenous cefoxitin, imipenem, or tigecycline, in addition to oral antibiotics (minocycline, clofazimine, moxifloxacin, linezolid; Floto et al., 2016). The impermeability of the cell envelopes of NTM to drugs and the high number of efflux systems and antibiotic inactivation mechanisms with which NTM are typically endowed confer upon these microorganisms high intrinsic protection against antibiotics (Brown-Elliott et al., 2012). There is clearly an urgent need for more active and better-tolerated drugs to improve therapeutic outcome (Jarand et al., 2011; Maurer et al., 2014; Park and Olivier, 2015; Martiniano et al., 2016).

In last 3 years, the phenotypic screening of compound libraries against NTM has yielded a number of hits with activity against MABSC, MAC, or both complexes. Interestingly, several of these compounds appear to kill NTM through the inhibition of MmpL3, an essential mycolic acid transporter present in all mycobacteria whose therapeutic potential in the treatment of *M. tuberculosis* infections was highlighted in a number of recent studies (Sacksteder et al., 2012; Kondreddi et al., 2013; Lun et al., 2013; Rao et al., 2013; Remuinan et al., 2013; Yokokawa et al., 2013; Li et al., 2016, 2017; Poce et al., 2016, 2018; Stec et al., 2016; Degiacomi et al., 2017). The availability of cidal inhibitors against this new target, some of which have already demonstrated activity in *in vivo* models of MABSC infection (Dupont et al., 2016; De Groote et al., in revision; Pandya et al., in revision), provides much-needed novel opportunities for the treatment of pulmonary NTM infections.

This perspective reflects on the therapeutic potential and promiscuity of MmpL3 in NTM, and discusses recent findings from our laboratories toward understanding the basis for the better activity and looser structure-activity relationship of MmpL3 inhibitors against *M. tuberculosis* compared to NTM.

## The phenotypic screening of compound libraries against NTM identifies inhibitors of MmpL3

In the last 3 years, the screening of compound libraries, including libraries of TB actives, against MABSC and MAC, has yielded a number of potent hits that appear to target the mycolic acid transporter MmpL3. These include indole-2-carboxamides (ICs) (Franz et al., 2017; Kozikowski et al., 2017; Low et al., 2017), benzothiazole amides (De Groote et al., in revision), and a piperidinol derivative (PIPD1) (Dupont et al., 2016; Low et al., 2017). Earlier work on analogs of the *M. tuberculosis* MmpL3 inhibitor BM212 had further highlighted the activity of pyrole derivatives against a variety of NTM including *M. avium, M. gordonae, M. smegmatis*, and *M. marinum* (Biava et al., 1999, 2007; Biava, 2002). A subset of these hits and their MIC against *M. tuberculosis, M. avium* and MABSC isolates (including *M. abscessus* subsp. *abscessus, M. abscessus* subsp. *Massiliense*, and *M. abscessus* subsp. *bolletti*) is presented in Table 1 along with that of other chemotypes reported to inhibit MmpL3 activity in *M. tuberculosis* (i.e., the 1,2-ethylene diamine SQ109, the tetrahydropyrazolopyrimide carboxamide THPP1, and the adamantyl urea AU1235) (Grzegorzewicz et al., 2012; La Rosa et al., 2012; Tahlan et al., 2012; Remuinan et al., 2013).

**Table 1:** MICs of MmpL3 inhibitors against *M. tuberculosis* [*Mtb*], *M. abscessus* complex species (*M. abscessus* subsp. *abscessus* ATCC 19977 [*Mabs*]; *M. abscessus* subsp. *massiliense* CIP 108297 [*Mmas*]; *M. abscessus* subsp. *bolletii* ATCC 14472 [*Mbol*]), *M. avium* 104 [*Mav*], and *M. smegmatis* recombinant strains expressing different *mmpL3* orthologs.

**Inhibitor**	**Structure**	***Mtb***	***Msmg***	***Mav***	***Mabs***	***Mmas***	***Mbol***	***Msmg***Δ***mmpL3***		
								***mmpL3smg***	***mmpL3abs***	***mmpL3tb***
PIPD1	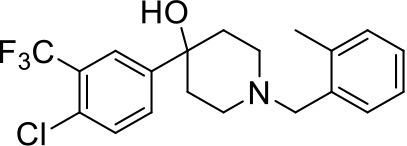	0.15^a^	<1	125^b^	0.125	0.125	nd	nd	nd	nd
AU1235	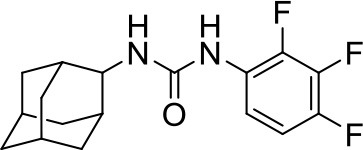	0.1–0.2	1.6–2.5	>32	0.5	1	0.5	2	2	0.3
BM212	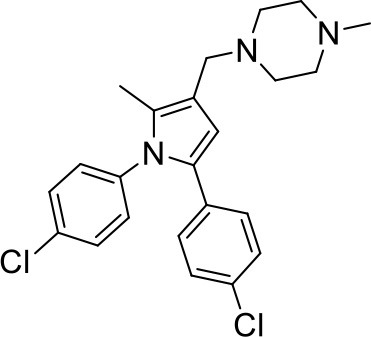	6	8–12	2	1–2	1–2	nd	8–12	8	4–6.2
SQ109	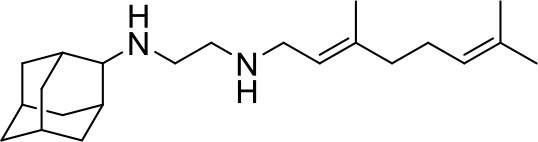	0.6-0.8	6.2–12.4	4	>32	>32	nd	8–12	16	0.4–0.8
THPP1	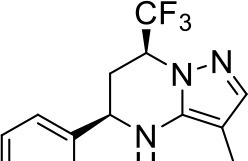	0.4-0.8	>25	>16	>16	>16	nd	>32	>32	0.8–2
NITD304	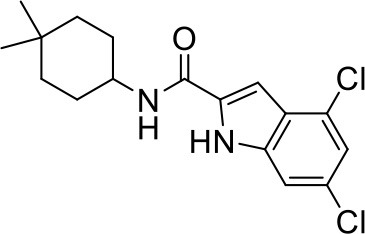	0.004	1	8	0.016	0.016	nd	1	0.12	0.06
NITD349	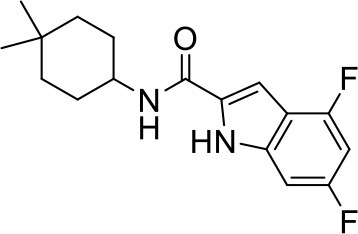	0.008	1	8	0.016	0.031	nd	0.25	0.25	0.06–0.12
IC5	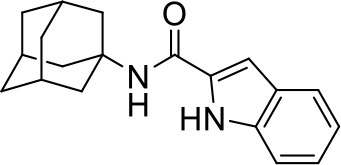	0.2	1.6–3.2	>32	0.25	0.5	0.25	1.6–3.2	3.2	0.2
IC6	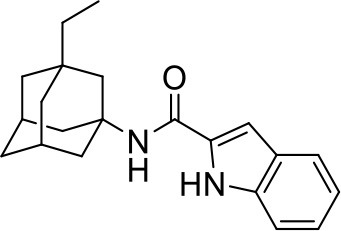	1.25	>20	>32	>32	>32	>32	>32	>32	1.25
IC9	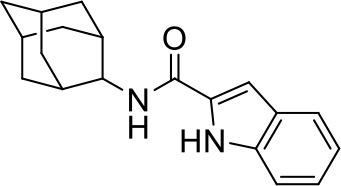	0.39	>25	>32	>32	>32	32	32	>32	0.25–0.39
IC10	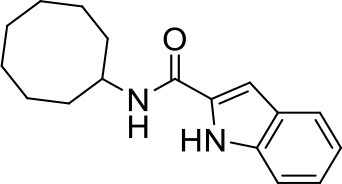	0.39	25	>32	>32	>32	32	25	>32	0.2
IC15	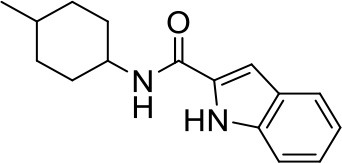	5	12.5	>32	>32	16	16	8–12.5	>32	3–4
IC16	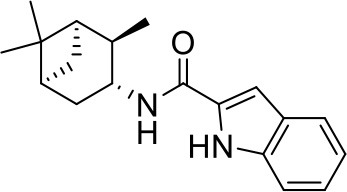	0.05	0.8	8	0.12	0.06	0.12	3–4	4	0.06
IC20	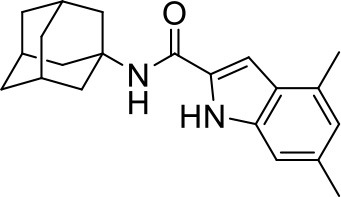	0.02	>20	>32	>32	>32	>32	>32	>32	0.16
IC21	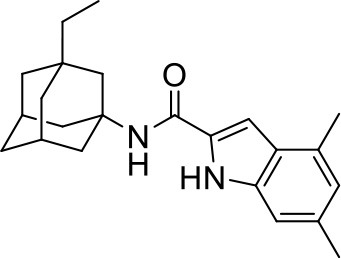	0.04	>20	>32	>32	> 32	>32	>32	>32	0.45
IC24	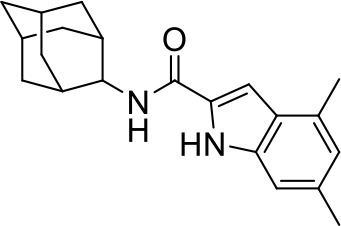	0.04	>20	>32	>32	>32	>32	>32	>32	0.16
IC25	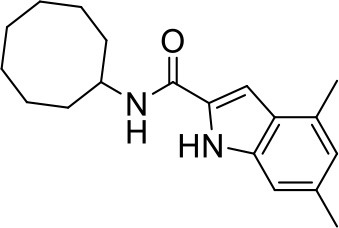	0.02	0.3–0.6	0.25–0.5	0.06	0.03	0.04	0.8–1	0.5	0.08
IC26	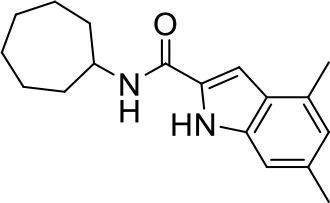	0.08	0.6	2	0.03	0.06	0.03	0.6–1	1	0.16–0.25
IC29	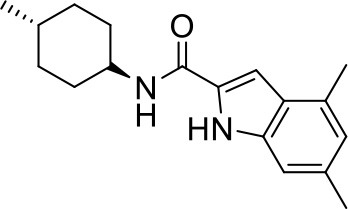	0.31	>20	>32	0.06	0.06	0.03	>32	1	0.62
IC30	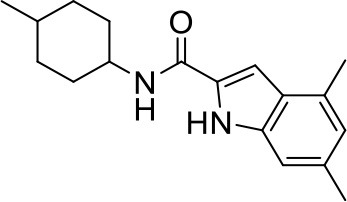	0.16	nd	>32	0.125	0.125	0.06	2	1	0.25–0.31
APRA	–	1	nd	2	2	4	nd	2	4	2
BDQ	–	0.5–1	nd	0.01	0.0625	nd	nd	<0.03	<0.03	<0.03
CFZ	–	0.5–1	nd	<0.125	0.125	nd	nd	1	1	0.5
CLA	–	<0.125		0.125	0.5-1	0.25	nd	0.5	1	0.5

*MICs (in μg/mL) were determined were determined in 96-well microtiter plates at 37°C in 7H9-ADC-0.05% Tween 80 medium (M. smegmatis), 7H9-OADC-0.05% tyloxapol supplemented with 0.2% casaminoacids, 48 μg/ml pantothenate, and 50 μg/ml L-leucine (M. tuberculosis mc^2^6206), cation-adjusted Mueller-Hinton broth (M. abscessus complex) or in cation-adjusted Mueller-Hinton broth supplemented with 5% OADC (M. avium) using the resazurin blue test (Martin et al., 2003) and by visually scanning for growth. The orthologs of mmpL3 from M. abscessus (mmpL3abs), M. tuberculosis (mmpL3tb), and M. smegmatis (mmpL3smg) are expressed from the replicative plasmid pMVGH1 under control of the hsp60 promoter in the background of a M. smegmatis null mutant (MsmgΔmmpL3) of which the entire mmpL3 ORF was deleted and replaced with a kanamycin-resistance cassette. Control drugs: APRA, apramycin; BDQ, bedaquiline; CFZ, clofazimine; CLA, clarithromycin. nd, not determined*.

a MIC value against M. tuberculosis H37Rv ATCC 27294 (Low et al., 2017);

b *The precise M. avium strain used by Dupont et al. (2016) in the MIC determination of PIPD1 was not indicated and may be different from M. avium 104*.

ICs have previously been identified as a novel chemical scaffold showing promise in the treatment of tuberculosis (Kondreddi et al., 2013; Lun et al., 2013; Rao et al., 2013; Stec et al., 2016). Based on their high anti-MABSC potency, bactericidal activity on extracellularly- and intracellularly-grown bacilli and promising safe pharmacological profile (Franz et al., 2017; Kozikowski et al., 2017; Pandya et al., in revision), two lead molecules were advanced for efficacy studies in a mouse model of MABSC infection. Oral administration of the lead compounds showed a statistically significant reduction in bacterial load in the lungs, spleen and liver of MABSC-infected mice compared to an untreated control group, with one of the two compounds (compound # IC25; see Table 1) showing similar efficacy to amikacin (Pandya et al., in revision). The intrapulmonary delivery of a lead benzothiazole amide compound also demonstrated *in vivo* efficacy in a mouse model of chronic MABSC lung infection (De Groote et al., in revision), whereas the piperidinol-based compound PIPD1 was reported to restrict bacterial growth in a zebrafish model of MABSC infection (Dupont et al., 2016). An interesting property of MmpL3 inhibitors first revealed in *M. tuberculosis* is their ability to synergize with a number of other antimycobacterial drugs or drug candidates including rifampicin, bedaquiline, clofazimine, and β-lactams (Li et al., 2017). Our preliminary studies with IC25 (see Table 1) in MABSC similarly point to the existence of a synergistic interaction between this MmpL3 inhibitor and clofazimine in MABSC (Table S1).

Collectively, these results highlight the therapeutic potential of MmpL3 inhibitors in the treatment of NTM infections and provide a strong incentive to develop these compounds into new generation antimycobacterial drugs as their inclusion in anti-NTM drug regimens has the potential to lead to the faster and more efficient clearance of NTM from infected tissues.

## MmpL3: a promiscuous mycobacterial target

The reason why so many chemical scaffolds kill *M. tuberculosis* and NTM through the inhibition of MmpL3 remains incompletely understood. MmpL3 belongs to the Resistance, Nodulation and Division (RND) superfamily of transporters that requires the transmembrane electrochemical proton gradient for activity. The observation that the most common resistance mutations identified in both *M. tuberculosis* and MABSC tend to map to a transmembrane region of MmpL3 overlapping with functional residues required for proton translocation or proton-driven conformational changes in the transporter has led to the hypothesis that inhibitors might target the proton relay site of MmpL3 (Belardinelli et al., 2016). MmpL3 inhibitors are typically lipophilic (logP ~2.6–7.0) and many suffer from poor aqueous solubility which likely favors their concentration in the inner membrane where MmpL3 is located. This property and the extreme vulnerability of MmpL3 (Li et al., 2016) that may allow inhibitors with relatively weak binding affinity to the transporter to still inhibit enough of its activity to cause growth arrest, could explain the bias of phenotypic screens toward small hydrophobic inhibitors of MmpL3. The exquisite vulnerability of MmpL3 may further mask potential secondary targets of the inhibitors as illustrated by THPP derivatives that were found to target another essential mycolic acid-related protein in *M. tuberculosis* (Cox et al., 2016) and compounds such as SQ109, BM212, and some THPPs that show activity against non-replicating *M. tuberculosis* bacilli, a property typically not shared by other MmpL3 inhibitors Li W. et al., 2014). The fact that the hydrophobicity of ICs, THPPs, SQ109 analogs and urea derivatives is a key driver of their efficacy provides further support to the notion that the concentration of MmpL3 inhibitors in the phospholipid bilayer plays a key role in their activity (Biava et al., 2005, 2007; Onajole et al., 2010, 2013; Brown et al., 2011; Scherman et al., 2012; Kondreddi et al., 2013; North et al., 2013; Li K. et al., 2014; Poce et al., 2016; Stec et al., 2016; Franz et al., 2017; Kozikowski et al., 2017).

A second mechanism through which high rates of apparent MmpL3 inhibitors may arise from phenotypic screens was proposed after it was found that unspecific uncouplers such as carbonyl cyanide *m*-chlorophenyl hydrazone (CCCP) or the K^+^ ionophore, valinomycin, both abolished MmpL3 activity in *M. tuberculosis* and *M. smegmatis* (Li W. et al., 2014). This finding indicated that any compound with the ability to dissipate the proton motive force may indirectly inhibit MmpL3 activity with immediate consequences on mycobacterial growth and viability. Accordingly, and most likely explaining the relatively broad spectrum of activity of some of these compounds including against bacteria devoid of MmpL3 homolog, inhibitors such as the 1,2-ethylenediamine SQ109, the adamantyl urea AU1235 and the 1,5-diarylpyrrole derivative BM212 were found to impact to some degree the membrane potential, the electrochemical proton gradient or both components of the proton motive force of mycobacterial cells (Li K. et al., 2014; Li W. et al., 2014; Feng et al., 2015; Foss et al., 2016). This unspecific activity, however, was later disputed in the case of BM212 and this compound proposed to directly inhibit MmpL3 on the basis of its demonstrated binding to the purified MmpL3 protein from *M. smegmatis* (Xu et al., 2017).

In conclusion, both direct and indirect mechanisms can lead to MmpL3 inhibition in treated mycobacterial cells and contribute to the promiscuity of the target. While not mutually exclusive, a precise understanding of how these two mechanism(s) play out for each inhibitor to eventually abolish mycolic acid export will require a detailed analysis of how each of them interacts with the transporter and affects the energy metabolism of the bacterium.

## NTM vs. *M. tuberculosis* efficacy

From the MIC data presented in Table 1 and previous studies (Biava et al., 1999, 2007; Biava, 2002; Li K. et al., 2014; Franz et al., 2017; Kozikowski et al., 2017; Low et al., 2017; De Groote et al., in revision), it is obvious that the overall activity of MmpL3 inhibitors against NTM, particularly MAC, is less than that observed against *M. tuberculosis*. While the structural diversity of the chemotypes found to inhibit MmpL3 is very broad, spanning from compounds such as BM212 and THPP1, which are large (for MmpL3 inhibitors) multicyclic compounds, to SQ109 which is an ethylene diamine originally designed as an ethambutol analog, the majority of MmpL3 inhibitors reported to date have come from two other classes that contain the same pharmacophore which are the ureas (e.g., AU1235) and indole-2-carboxamides. The pharmacophore for these classes of MmpL3 inhibitors are two hydrogen bond donors and one hydrogen bond acceptor in the center of the molecule and one bulky lipophilic aliphatic ring (adamantyl, cyclooctyl, cycloheptyl, or substituted cyclohexyl groups) and one aromatic ring on either side of the core. For structure activity relationships, generally, as lipophilicity is increased on either the bulky aliphatic ring (typically through ring expansion or addition of methylene or methyl groups) or aromatic ring (typically through addition of halogens or methyl groups), anti-NTM activity is improved.

Since a number of factors could account for the overall better activity of MmpL3 inhibitors against *M. tuberculosis* than NTM, including species-specific variations in the structure of MmpL3 orthologs, increased drug efflux/degradation/modification in NTM relative to *M. tuberculosis*, or reduced compound penetration in NTM, we first sought to compare the MIC of the inhibitors presented in Table 1 against *M. smegmatis* recombinant strains expressing different MmpL3 orthologs. To this end, *mmpL3* from *M. tuberculosis, M. smegmatis* and *M. abscessus* subsp. *abscessus* were expressed from the same expression plasmid in the background of a *M. smegmatis* mutant whose endogenous *mmpL3* gene was deleted by allelic replacement (*Msmg*Δ*mmpL3*) (Belardinelli et al., 2016). Expressing all orthologs from the same promoter in the same *M. smegmatis* strain abolished any potential differences in compound uptake, modification and efflux allowing for a direct comparison of the effect of the inhibitors against the three MmpL3 proteins. Importantly, all three *mmpL3* orthologs were expressed at comparable levels in this recombinant system (Figure S1). The results of these comparative MIC studies clearly indicated that the MICs of the inhibitors against the different *M. smegmatis* strains generally reflected their MICs against the *Mycobacterium* species from which the rescue *mmpL3* ortholog originated (Table 1). The structure of MmpL3 thus appears to be the main driver of the susceptibility of each *Mycobacterium* species to these inhibitors.

## MmpL3 modeling

**Figure 1:**
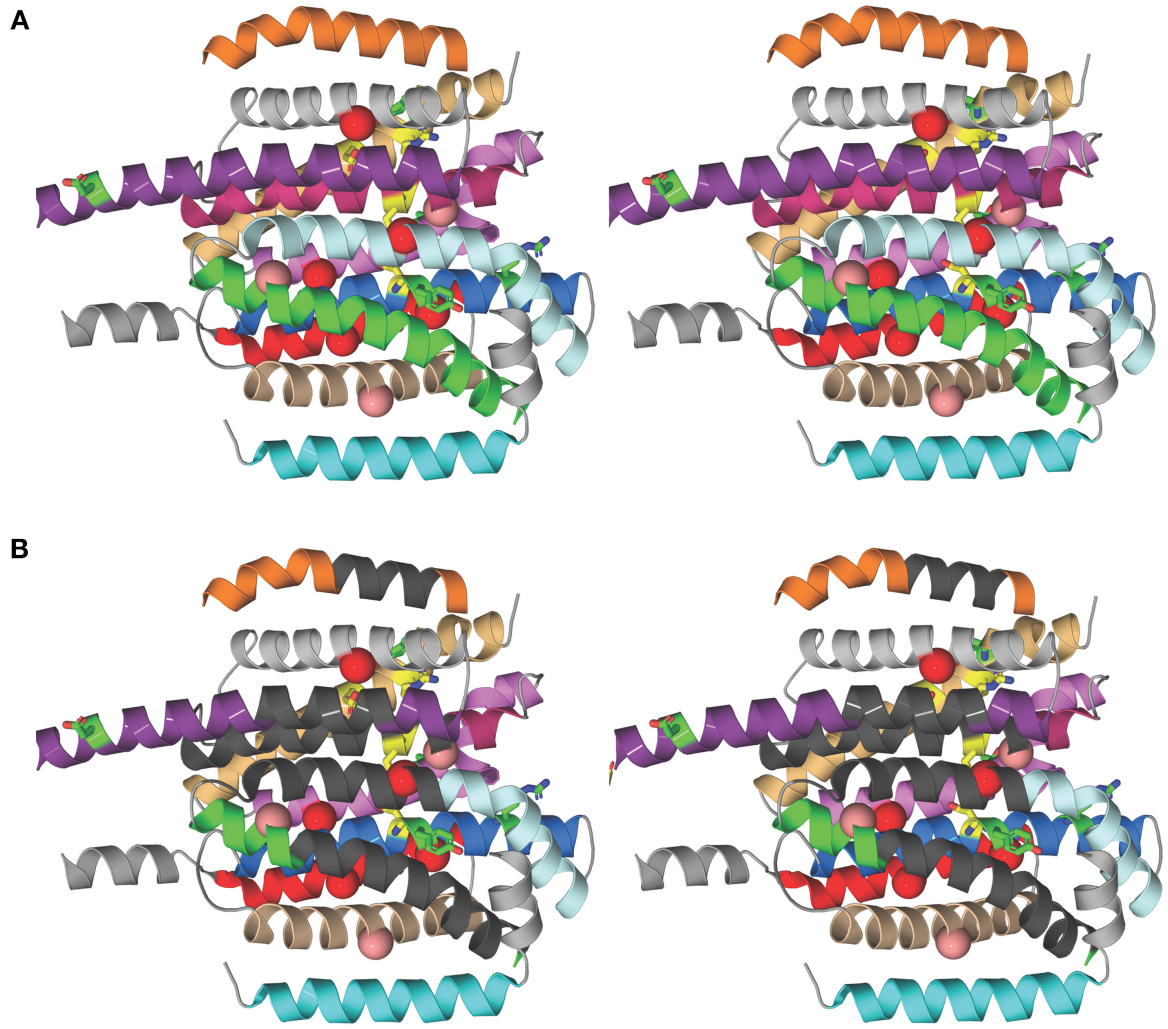
Structural comparison of NTM and *M. tuberculosis* MmpL3 transporters. **(A)** Stereo model showing the transmembrane (TM) helices of the *M. tuberculosis* MmpL3 subunit structure as predicted by I-TASSER. The TM helices are color-coded to improve visibility. From top to bottom, TMS-7 (orange), TMS-9 (gray), TMS-8 (violet purple), TMS-10 (pink), TMS-12 (light orange), TMS-11 (violet), TMS-5 (pale cyan), TMS-4 (marine), TMS-6 (green), TMS-2 (red), TMS-3 (wheat), and TMS-1 (cyan). The TM helices encompass residues whose mutations resulted in significant reduction in transport activity, shown in green, and residues 251, 288, 640, 641, 710, and 715 whose mutation abolished transport activity, colored yellow (Belardinelli et al., 2016). The positions of frequently encountered resistance mutations to one or more MmpL3 inhibitor series are shown as red and salmon spheres centered on the Cα atom of the native MmpL3 residue (Belardinelli et al., 2016). These residues map to TM helices. Resistance mutations also producing a significant reduction in growth are shown in red and those that slightly attenuated growth are colored salmon. **(B)** Stereo model as in **(A)** showing regions of the TM helices where the majority of residues are not conserved between MmpL3 orthologs (dark gray). Most of the dissimilar residues represent semi-conservative and non-conservative mutations (see Figure S2). Several of these regions map vicinal to the functional residues and mutations that induce resistance.

To investigate the structural basis for the different susceptibilities of MmpL3 orthologs to various classes of inhibitors, the I-TASSER server (Yang et al., 2015) was used for automated full-length 3D structure prediction of MmpL3 transporters from *M. tuberculosis* H37Rv, *M. abscessus* ATCC 19977, *M. smegmatis* mc^2^155, and *M. avium* 104. The top predicted structure for each MmpL3 transporter corresponded to a C-score of >-1.5 suggesting a correct fold. All MmpL3 orthologs resemble each other (root mean square differences based C-alpha atoms < 0.4Å) and had as closest target the crystal structure of the *Burkholderia multivorans* hopanoid transporter HpnN (PDB 5khnB). Comparison of the predicted structures with that of HpnN yielded a high TM-score >0.8 and low RMSD <2.0 between residues that were structurally aligned by TM-align (Zhang, 2008). The superposition of all three NTM MmpL3 orthologs onto MmpL3 from *M. tuberculosis* H37Rv shows very similar spatial overlap of the C-alpha positions of essential residues identified in the reference MmpL3 transporter (Belardinelli et al., 2016) that can be seen in Figure 1A. Each of the predicted structures contained 12 TM helices.

We next aligned the amino acid sequences of the MmpL3 orthologs among each other using PSI/TM-Coffee (Chang et al., 2012; Figure S2). Essential functional residues identified in *M. tuberculosis* MmpL3 (namely, residues: 251, 288, 640, 641, 710, and 715; boxed in green in Figure S2; Belardinelli et al., 2016) are conserved and are all located in the central regions of the 12 TM helical bundle that is thought to be involved in proton translocation.

We then searched for sequence dissimilarities among the TM helices given the lipophilicity of the MmpL3 inhibitors. The stereo model in Figure 1B shows regions of the transmembrane helices in dark gray where the majority of amino acid residues are not conserved between MmpL3 orthologs and which span the central regions of the 12 TM helical bundle. Most of the dissimilar residues represent semi-conservative and non-conservative mutations that are shown as red boxes on the sequence alignment shown in Figure S2. Several of these regions map vicinal to the functional residues and mutations that induce resistance. The shape differences in the geometries of the hydrophobic and polar side chains alters the packing of the 12 TM helices and is likely to concomitantly modify their dynamical behavior important for transport activity and inhibitor binding. Given that the inhibitors are partitioned into the lipid bilayer, from a thermodynamic perspective, their propensity to interact with the TM helices will further depend on two factors: their ability to interact preferentially with the hydrophobic side chains of the TM helices and their ability to form polar interactions with either backbone or polar side chains. It follows that both the differential helical packing modifying the binding loci of the inhibitors and the nature of the side chains of the TM helices probably account for the ortholog-dependent activity of MmpL3 inhibitors.

## Future directions

There is an unmet medical need for the development of new bactericidal agents to treat pulmonary NTM infections. The novel classes of bactericidal MmpL3 inhibitors that have been reported in the last few years, some of which have demonstrated activity against *M. tuberculosis* and MABSC *in vivo*, highlight the therapeutic potential of this transporter in tuberculous and nontuberculous mycobacteria and provide much needed translational opportunities for the treatment of NTM infections. Future research is expected to gain further insight into the structure of MmpL3 and its variations across *Mycobacterium* species in order to leverage the emerging structure-activity relationship information now available for some of these compound series (Brown et al., 2011; Scherman et al., 2012; North et al., 2013; Li K. et al., 2014; Poce et al., 2016, 2018; Stec et al., 2016; Franz et al., 2017; Kozikowski et al., 2017). Also, critical to the further development of these inhibitors will be the availability of a simple, non-radioactive, and relatively high-throughput assay to screen optimized analogs with increased activity against MmpL3. Currently available cell-free and whole cell-based assays (e.g., Grzegorzewicz et al., 2012; Li W. et al., 2014; Li et al., 2016; Xu et al., 2017) indeed lack the simplicity of use and/or specificity required to rapidly screen such analogs. The development of such assays is currently the object of intense efforts in our laboratories.

## Author contributions

EN, JS, and MJ conceived the project, analyzed the data, and wrote the manuscript. WL, PH, WT, and VC generated and characterized the *M. smegmatis* recombinant strains, and carried out the MIC determinations and checkerboard assays. AP synthesized inhibitors, contributed to the preparation of Table 1 and analyzed SAR data. AY performed the MmpL3 modeling studies.

### Conflict of interest statement

The authors declare that the research was conducted in the absence of any commercial or financial relationships that could be construed as a potential conflict of interest.
