# Supplementary material for: MmpL3 as a Target for the Treatment of Drug-Resistant Nontuberculous Mycobacterial Infections
Source: Front Microbiol. 2018 Jul 10;9:1547. doi: 10.3389/fmicb.2018.01547 (PMC6048240; doi:10.3389/fmicb.2018.01547)

Supplementary Material

MmpL3 as a target for the treatment of drug-resistant nontuberculous mycobacterial infections

Wei Li[1#], Amira Yazidi[2,3#], Amitkumar N. Pandya[4#], Pooja Hegde[4], Weiwei Tong[1], Vinicius Calado Nogueira de Moura[1], E. Jeffrey North[4*], Jurgen Sygusch[2,3*] and Mary Jackson[1*]

[1]Mycobacteria Research Laboratories, Department of Microbiology, Immunology and Pathology, Colorado State University, Fort Collins, Colorado, USA; [2]Biochimie et Médecine Moléculaire, Université de Montréal, Montréal, Quebec, Canada; [3]Groupe d'Étude des Protéines Membranaires (GÉPROM), Université de Montréal, Montréal, Quebec, Canada; [4]Department of Pharmacy Sciences, School of Pharmacy and Health Professions, Creighton University, Omaha, Nebraska, USA

# Co-first authors
* Co-corresponding authors

Correspondence: Dr. E. Jeffrey North (jeffreynorth@creighton.edu); Dr. Jurgen Sygusch (jurgen.sygusch@umontreal.ca); Dr. Mary Jackson (Mary.Jackson@colostate.edu)

Table S1: Interaction of indole-2-carboxamides with other antimycobacterial drugs and experimental compounds against *M. abscessus subsp. massiliense* CIP 108297 as determined by REMA checkerboard.

Five combinations with the indole-2-carboxamide compound IC25 were tested against *M. abscessus subsp. massiliense* CIP 108297. Bacterial cultures were grown for 3 days at 37°C in Middlebrook 7H9 broth supplemented with 10 % ADC (BD, Difco) and 0.05 % Tween 80 before the addition of resazurin. MIC values by REMA (in µg/ml) were read after an additional overnight incubation at 37°C.

The fractional inhibitory index (ΣFIC) of each drug combination was calculated as described previously [Odds, 2003]. ΣFIC values ≤ 0.5 indicate synergistic activity; values ≥ 4 indicate antagonism; and values in between correspond to additivity (no interaction).

APRA, apramycin; AZI, azithromycin; BDQ, bedaquiline; CFZ, clofazimine; CLA, clarithromycin.

		Interaction with IC25	
Compound	MIC	ΣFIC	outcome
IC25	0.05	-	-
CFZ	1	0.5	synergistic
BDQ	0.125	2	additive
AZI	2	1.12	additive
CLA	0.125	0.75	additive
APRA	2	1	additive

Reference:

Odds, F. C. (2003) Synergy, antagonism, and what the chequerboard puts between them. J. Antimicrob. Chemother. 52, 1.

<u>Figure S1</u>: Level of expression of the *M. smegmatis*, *M. tuberculosis* and *M. abscessus* MmpL3 orthologs in *Msmg*Δ*mmpL3*.

The level of expression of the three *mmpL3* orthologs in *Msmg*Δ*mmpL3* was compared by qRT-PCR using primers mmpL3_O1 (5'-GCGCGCTGGGCATCATGC-3') and mmpL3_O2 (5'-GCGGAACCGGCTCACGATG-3'). The target cDNA was normalized internally to the *sigA* cDNA in the same sample. mRNA levels are means ± standard deviations of two PCR reactions performed on two cDNA preparations. Student's *t*-test analysis showed no statistically significant differences between *mmpL3* orthologs ($p > 0.05$).

RNA was extracted from 5-ml cultures grown to an OD_{600} of 0.6 using the Direct-zol™ RNA Miniprep kit (Zymo Research) per the manufacturer's instructions. Reverse transcription reactions were carried out using the SuperScript™ IV First-Strand Synthesis System (Invitrogen), and qRT-PCRs were run using the PerfeCTa SYBR Green Fast Mix (Quanta BioSciences). PCR conditions: 95°C (2 min; enzyme activation), followed by 40 cycles of 95°C (15 sec; denaturation) and 60°C (30 sec; annealing/extension). Mock reactions (no reverse transcription) were done on each RNA sample to rule out DNA contamination.

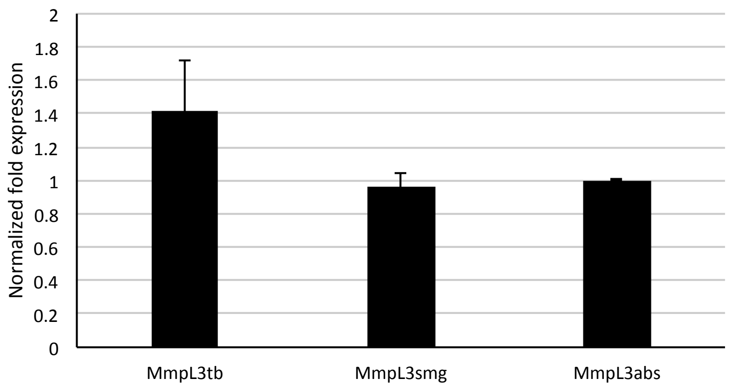

<u>Figure S2</u>: Amino acid sequence alignment of MmpL3 from *M. tuberculosis*, *M. abscessus* ATCC 19977, *M. avium* 104 and *M. smegmatis* mc^{2}155 using PSI/TM-Coffee.

Conserved sequence (*), conservative mutations (:), semi-conservative mutations (.), and non-conservative mutations ( ). Essential functional residues are boxed in green. Regions of dissimilarity between MmpL3 orthologs are shown as red boxes.

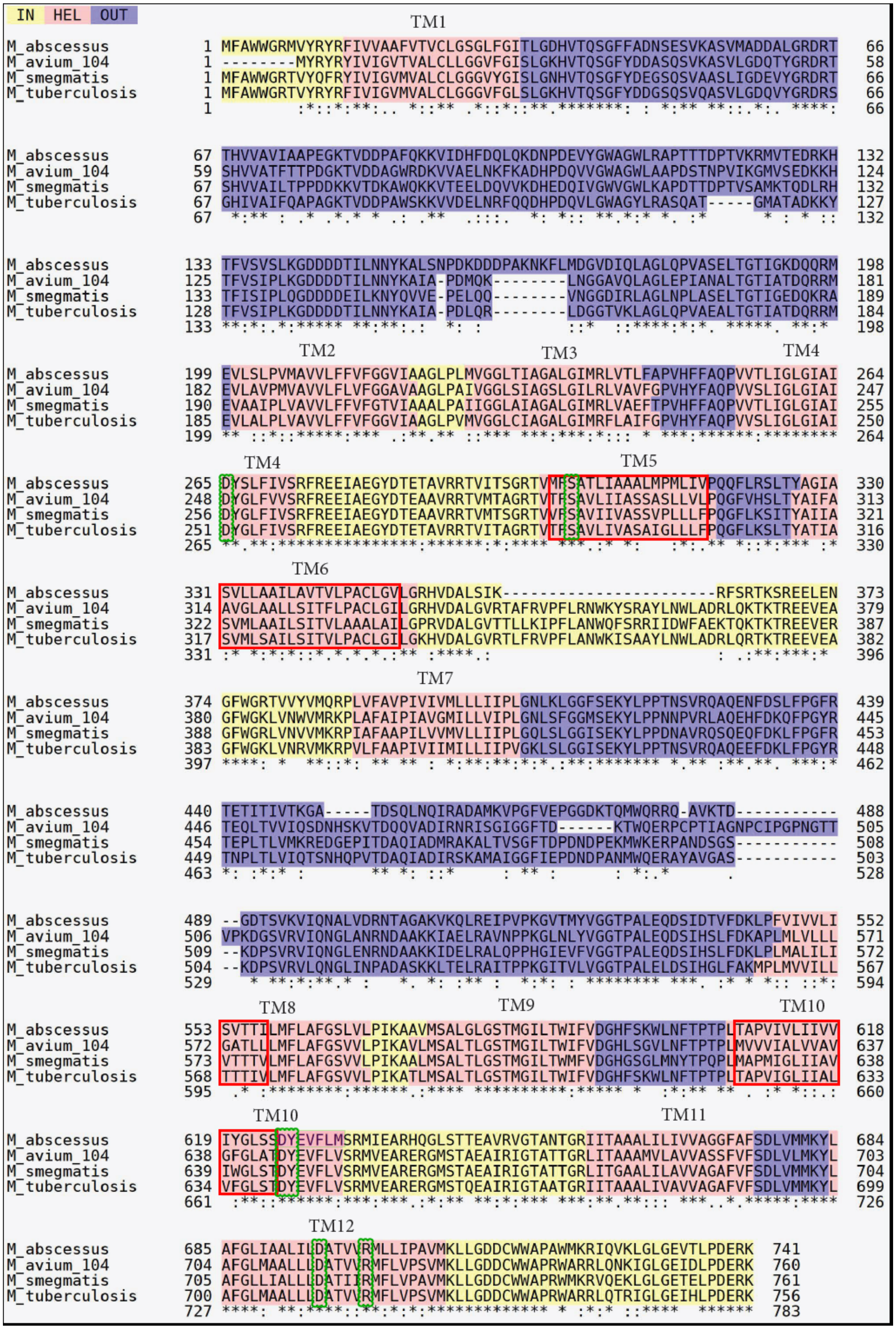

Supplement: Supplementary file 1 [file Presentation_1.PDF]
